# Repression of phosphoinositide-dependent protein kinase 1 expression by ciglitazone via Egr-1 represents a new approach for inhibition of lung cancer cell growth

**DOI:** 10.1186/1476-4598-13-149

**Published:** 2014-06-13

**Authors:** SWei Sunny Hann, Qing Tang, Fang Zheng, Shunyu Zhao, Jianping Chen, ZhiYu Wang

**Affiliations:** 1 University of Guangzhou Traditional Chinese Medicine, Guangdong Academy of Traditional Chinese Medicine, Guangdong Provincial Hospital of Chinese Medicine, Guangzhou, Guangdong Province, China 510120; 2 School of Chinese Medicine, Li Ka Shing Faculty of Medicine, University of Hong Kong, 10 Sassoon Road, Hong Kong, China 00852

**Keywords:** Human lung adenocarcinoma cell, Egr-1, PDK1, Ciglitazone, AMPKα, Metformin

## Abstract

**Background:**

Peroxisome proliferator-activated receptors gamma (PPARγ) ligands have been shown to inhibit the growth of non-small cell lung cancer (NSCLC) cells. However, the mechanisms underlying this effect remain incompletely elucidated.

**Methods:**

Cell proliferation and apoptosis were measured by cell viability, MTT and caspase3/7 activity assays. Phosphorylation/protein expression and gene silence/overexpression of AMPKα, phosphoinositide-dependent protein kinase 1 (PDK1), Egr-1 and PPARγ were performed by Western blot and siRNA/transfection assays. Dual-Luciferase Reporter Kit was used to measure the PPAR response elements (PPRE) reporter and PDK1 promoter activities, and ChIP assay was used to detect the Egr-1 protein binding to the DNA site in the *PDK1* gene promoter.

**Results:**

We found that ciglitazone, one synthetic PPARγ ligand, inhibited growth and induced apoptosis of NSCLC cells through decreased expression of PDK1, which was not blocked by GW9662 (a specific PPARγ antagonist). Overexpression of PDK1 overcame the effect of ciglitazone on cell growth and caspase 3/7 activity. Ciglitazone increased the phosphorylation of AMPKα and c-Jun N-terminal kinase (JNK), and the inhibitor of AMPK (compound C), but not JNK (SP600125), reversed the effect of ciglitazone on PDK1 protein expression. Ciglitazone reduced *PDK1* gene promoter activity, which was not observed in cells exposed to compound C, but not silenced of PPARγ siRNA. Combination of ciglitazone and metformin further reduced PDK1 expression and promoter activity. Furthermore, we showed that ciglitazone induced the protein expression of Egr-1, which was not observed in cells silencing of AMPKα. Moreover, silencing of Egr-1 abrogated the effect of ciglitazone on PDK1 promoter activity and cell growth. On the contrary, overexpression of Egr-1 enhanced the effect of ciglitazone on *PDK1* gene promoter activity. ChIP assays demonstrated that ciglitazone induced Egr-1 protein bind to the specific DNA site in the *PDK1* gene promoter.

**Conclusion:**

Collectively, our results demonstrate that ciglitazone inhibits PDK1 expression through AMPKα-mediated induction of Egr-1 and Egr-1 binding to the specific DNA site in the *PDK1* gene promoter, which is independent of PPARγ. Activation of AMPKα by metformin enhances the effect of ciglitazone. In turn, this leads to inhibition of NSCLC cell proliferation.

## Background

Lung cancer remains the leading cause of cancer-related mortality in the United States, and 30% to 40% of newly diagnosed patients with non-small cell lung cancer (NSCLC) present with regionally advanced and unresectable stage III disease [1]. Despite recent advances in understanding the molecular biology of lung cancer and the introduction of multiple new chemotherapeutic agents for its treatment, the poor outcomes related to lung cancer have not changed substantially [2,3]. This justifies the continuing search for agents with therapeutic potential against NSCLC.

Peroxisome proliferator-activated receptors (PPARs isotypes α, β/δ,γ) are ligand-inducible nuclear transcription factors that heterodimerize with retinoid X receptors and bind to PPAR response elements (PPRE) located in the promoter region of PPAR target genes [4]. The role of PPARγ, one PPAR isotype, has been extensively studied thanks to the availability of synthetic PPARγ agonists including antidiabetic drugs, such as rosiglitazone, ciglitazone, and pioglitazone [5]. These drugs are also effective in regulating cell activation, differentiation, proliferation, and apoptosis through both PPARγ-dependent and -independent signaling [6,7]. However, the detailed mechanisms responsible for these effects remain incompletely elucidated.

Stress-activated protein kinase/c-Jun N-terminal kinase (SAPK/JNK) is a mitogen-activated protein kinase family member that is activated by diverse stimuli and plays a critical role in regulating cell fate, being implicated in a multitude of diseases ranging from cancer to neurological, immunological and inflammatory conditions. JNK signaling is required for normal mammary gland development and has a suppressive role in mammary tumorigenesis [8]. AMP-activated protein kinase (AMPK), a heterotrimeric protein complex with serine/threonine kinase activity, has been involved in the regulation of a number of physiological processes including β-oxidation of fatty acids, lipogenesis, protein and cholesterol synthesis, as well as cell cycle inhibition and apoptosis. AMPK has been shown to act upstream and downstream of known tumor suppressors. However, whether AMPK acts as a *bona fide* tumor suppressor or a oncogene and, of particular importance, if AMPK should be targeted for activation or inhibition during cancer therapy, is controversial [9]. Early growth response-1 (Egr-1) is a Cys2-His2-type zinc-finger transcription factor. A broad range of extracellular stimuli is capable of activating Egr-1, thus mediating growth, proliferation, differentiation or apoptosis. Egr-1 is, therefore, participating in the progression of a variety of diseases such as atherosclerosis or cancer. A growing body of evidence suggests that Egr-1 functions as a tumor suppressor [10-12].

In an effort to explore the anti-tumor effects of ciglitazone on potential targets, we turned our attention to 3-phosphoinositide-dependent protein kinase 1 (PDK1), a master regulator of signal cascades that is involved in suppression of apoptosis and promotion of tumor growth including lung cancer [13]. Reduction of PDK1 by small interfering RNA (siRNA) in several cancer cells results in significant growth inhibition [14-17]. These observations suggest that PDK1 can be used as a target for cancer therapies.

Here, we report that ciglitazone inhibits NSCLC proliferation by inhibiting PDK1 expression through activation of AMPKα and induction of Egr-1 that is independent of PPARγ.

## Results

### Ciglitazone decreased growth and induced apoptosis in lung cancer cells, and inhibited PDK1 protein expression independent of PPARγ

**Figure 1:**
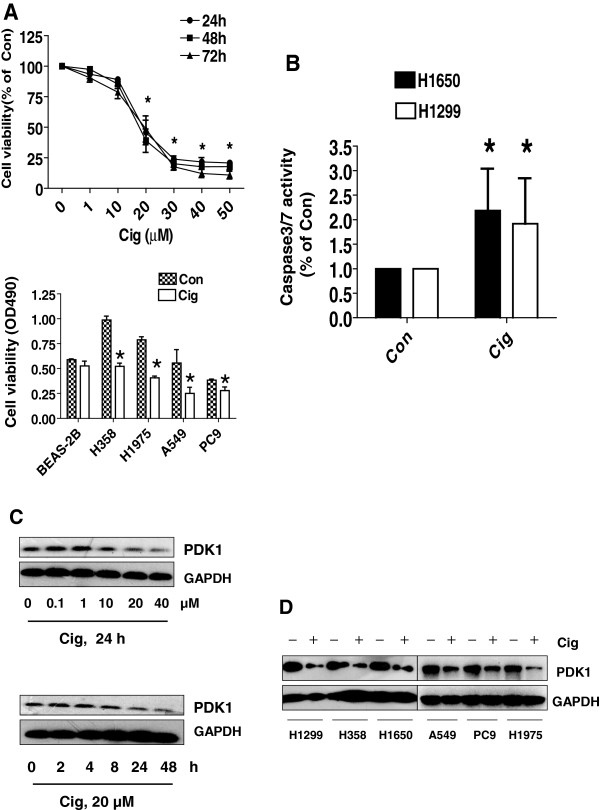
**Ciglitazone decreased growth and induced apoptosis in lung cancer cells. A**, H1299 cells were treated with increased concentrations of ciglitazone for up to 72 h (upper panel). NSCLC cells indicated were treated with ciglitazone (20 μM) or up to 48 h (lower panel). The cell viability was determined using the MTT assay as described in the Materials and Methods section in three separate experiments. **B**, Caspase 3/7 activity assay was performed on H1299 cells treated with or without ciglitazone for 48 h. Relative caspase 3/7 activity is indicated as percentage of untreated control cells. Results represent those obtained in three experiments. *indicates significant difference as compared to the untreated control group (P < 0.05). **C**, Cellular protein was isolated from H1299 cells that were cultured with increased concentrations of ciglitazone for up to 24 h (upper), or with ciglitazone (20 μM) for indicated period of time (lower), followed by Western blot. **D**, Cellular protein were isolated from NSCLC cells (H1299, PC9, A549, H1957, H358 and H1650) that were cultured with ciglitazone (20 μM) for up to 24, followed by Western Blot.

**Figure 2:**
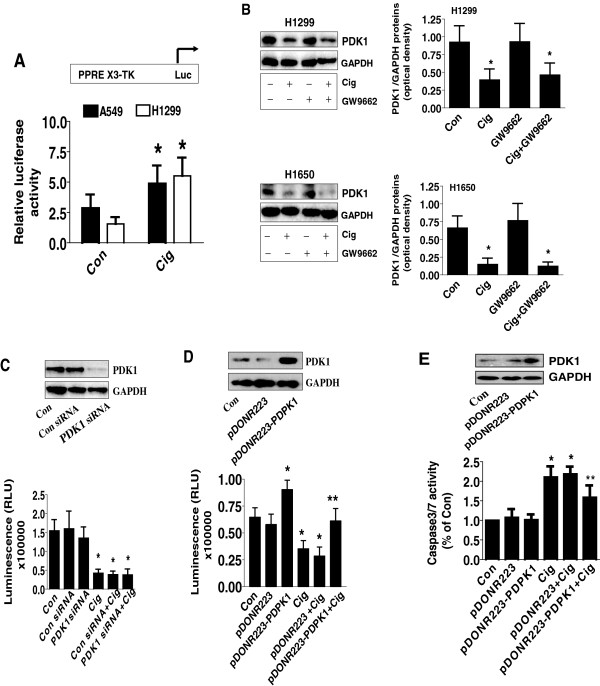
**Ciglitazone inhibited PDK1 protein expression independent of PPARγ. A**, H1299 and H1650 cells were transfected with control or PPRE X3-TK-luc reporter (from Addgene) for 24 h, followed by treating with ciglitazone for an additional 24 h. Afterwards, the Luciferase reporter activity was measured using Luciferase Assay System (Promega) according to manufacturer's instructions. The bars represent the mean ± SD of at least three independent experiments for each condition. *indicates significant difference as compared to the untreated control group (P < 0.05). **B**, Cellular protein was isolated from H1299 and H1650 cells cultured for 1 h in the presence or absence of GW9662 (20 μM) before exposing the cells to ciglitazone (20 μM) for an additional 24 h, then subjected to Western blot analysis. **C**, H1299 cells were transfected with control or PDK1 siRNA (80 nM) for 40 h, followed by exposing the cells to ciglitazone (20 μM) for an additional 24 h. Afterwards, the luminescence of viable cells was detected using Cell Titer-Glo Luminescent Cell Viability Assay kit. **D-E**, H1299 cells were transfected with the control and PDK1 expression vectors using the oligofectamine reagent according to the manufacturer’s instructions. After 24 h of incubation, cells were treated with or without ciglitazone for an additional 24 h. Afterwards, the luminescence of viable cells was detected using Cell Titer-Glo Luminescent Cell Viability Assay kit **(D)**. In separate experiment, the relative caspase 3/7 activity **(E)** is indicated as percentage of untreated control cells. The bars represent the mean ± SD of at least four independent experiments for each condition. Insert on the top panel shows a Western blot for PDK1 protein. *indicates significant difference as compared to the untreated control group (P < 0.05). **Indicates significance of combination treatment as compared with ciglitazone alone (P < 0.05).

We first examined the effect of ciglitazone on growth and apoptosis of lung cancer cells. We found that ciglitazone inhibited growth of lung cancer cell H1650 in the time- and dose-dependent manner, with significant inhibition observed at 20 μM at 48 h (Figure 1A, upper panel). Similar results were also observed in other NSCLC cell lines (Figure 1A, lower panel). We also showed that ciglitazone induced caspase 3/7 activity in H1650 cells indicating increase in apoptosis (Figure 1B). We then examined whether ciglitazone affected the expression of PDK1. We found that ciglitazone inhibited PDK1 protein expression in a time- and dose-dependent manner, with an effective response of 20 μM at 24 h in H1650 cells (Figure 1C). Reduction of PDK1 protein expression by ciglitazone was also found in other NSCLC cell lines (Figure 1D).We then tested whether the effects of ciglitazone on PDK1 were mediated through the activation of PPARγ. We showed that, while ciglitazone increased the PPRE luciferase activity (activation of PPAR) (Figure 2A), the effects of ciglitazone on PDK1 expression were not eliminated in the presence of GW9662, a specific PPARγ antagonist (Figure 2B) and in cells (H1299 and H1650) silencing of PPARγ (not shown). The result suggests that PPARγ-independent signals mediate the effect of ciglitazone on PDK1 protein expression.

Next, to test whether ciglitazone affects cell growth through PDK1-mediated signals, we blocked the PDK1 gene using PDK1 siRNA. We showed that knockdown of PDK1 significantly reduced PDK1 production, while the control siRNA had no effect (Figure 2C, upper panel). Cells exposed to PDK1 siRNA showed a slight reduction in cell proliferation at baseline; however, they showed significant reduction in growth in the presence of ciglitazone as determined by cell viability assay (Figure 2C, lower panel). Overexpression of PDK1 has been reported to correlate with tumor progression [15]. We found that overexpression of PDK1 abrogated the effect of ciglitazone on cell growth (Figure 2D, lower panel) and caspase 3/7 activity (Figure 2E). Transfection with PDK1 expression vector was confirmed by Western blot (Figure 2D-E, upper panel). Together, this suggested that ciglitazone not only inhibited growth but also increased apoptosis of lung cancer cells through, at least in part, the inhibition of PDK1.

### The role of AMPKα and SAPK/JNK in mediating the effect of ciglitazone on PDK1 protein expression

**Figure 3:**
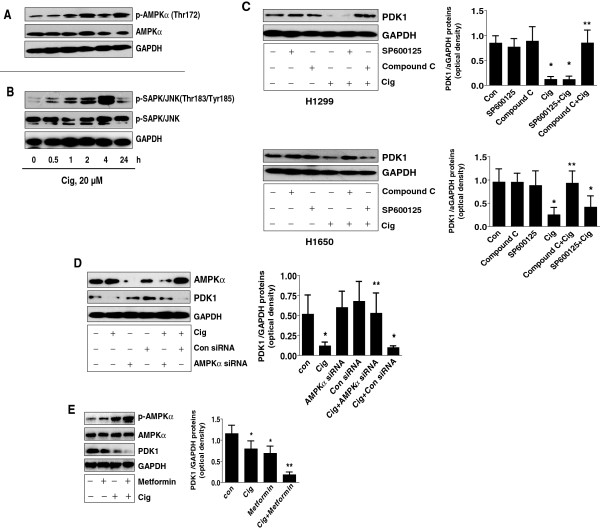
**The role of AMPK and SAPK/JNK in mediating the effect of ciglitazone on PDK1 protein expression. A-B**, Cellular protein were isolated from H1299 cells that were cultured with ciglitazone for up to 24, followed by Western Blot for phosphor-AMPKα, SAPK/JNK and total AMPKα, SAPK/JNK. **C**, Cellular protein was isolated from H1299 and H1650 cells treated with SP600125 (10 μM) or compound C (20 μM) for 1 h before exposure of the cells to ciglitazone for an additional 24 h. Afterwards, Western blot was performed. **D**, Cellular protein was isolated from H1299 cells transfected with control or AMPKα siRNA (80 nM) for 40 h, followed by exposing the cells to ciglitazone for an additional 24 h. Afterwards, Western blot was performed. **E**, Cellular protein was isolated from H1299 cells treated with ciglitazone and metformin (5 mM) for 24 h, followed by Western Blot. GAPDH served as internal controls for normalization purposes. The bar graph represents the mean ± SD of PDK1/GAPDH of at least three independent experiments. *Indicates significant difference from untreated control. **Indicates significance of combination treatment as compared with ciglitazone alone (P < 0.05).

Studies by this group and others also demonstrated a role for AMPK in mediating the effect of PPARγ ligands, such as thiazolinediones (TZDs) compounds, in different cell systems [18,19]. We showed that ciglitazone increased phosphorylation of AMPKα and SAPK/JNK with maximal effect observed at 2–4 h in H1650 cells (Figure 3A-B). Interestingly, the inhibitors of AMPK, compound C, but not of SAPK/JNK, SP600125, blocked the inhibitory effect of ciglitazone on PDK1 protein expression in both H1650 and H1299 cells (Figure 3C). Similarly, silencing of AMPKα abrogated the effect of ciglitazone on PDK1 protein (Figure 3D). This indicates the specificity of AMPKα activation in this process. Interestingly, combination treatment of ciglitazone and metformin, an activator of AMPK, further reduced the PDK1 protein expression (Figure 3E).

### Ciglitazone decreases PDK1 promoter activity independent of PPARγ activation

**Figure 4:**
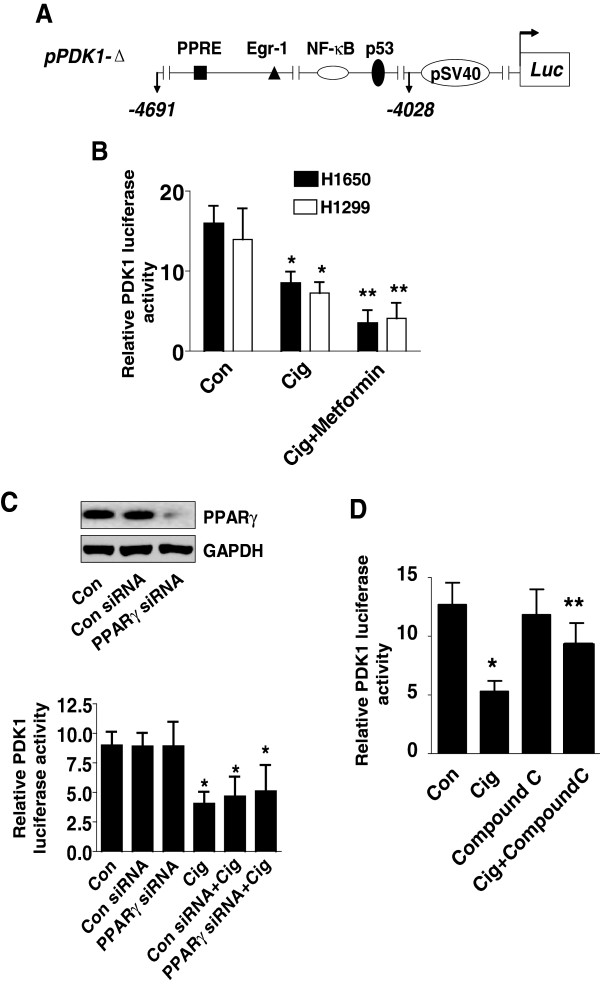
**Ciglitazone decreased PDK1 promoter activity. A**, The human PDK1 wild type reporter construct schematics are presented. These regions contain several transcription factor binding sites including PPRE, Egr-1, p53 and NF-κB. **B**, H1299 and H1650 lung cancer cells (1 × 10^5^ cells) were transfected with a wild type human PDK1 promoter reporter construct ligated to luciferase reporter gene and an internal control Renilla Luciferase Reporter Vector for 24 h. Afterward, cells were treated with ciglitazone for an additional 24 h. **C**, H1299 cells were transfected with control or PPARγ siRNAs (80 nM) together with a wild type ILK promoter construct for 30 h, then cells were exposed to ciglitazone for an additional 24 h. Insert on the top shows Western blot result for PPARγ protein. GAPDH served as internal control for normalization purposes. **D**, H1299 cells (1 × 10^5^ cells) were transfected with a wild type human PDK1 promoter reporter construct ligated to luciferase reporter gene and an internal control Renilla Luciferase Reporter Vector as described in Materials and Methods for 24 h. Afterwards, cells were treated with SP600125 (10 μM) for 1 h before exposure of the cells to ciglitazone for an additional 24 h. The ratio of firefly luciferase to renilla luciferase activity was quantified as described in Material and Methods. The bars represent the mean ± SD of at least four independent experiments for each condition. *Indicates significant increase of activity as compared to controls. **Indicates significance of combination treatment as compared with ciglitazone alone (P < 0.05).

We also examined if the effects of ciglitazone on PDK1 expression occurred at the transcriptional level. As shown in Figure 4A, the *PDK1* gene promoter contains multiple transcription factor binding sites including PPRE, Egr-1, nuclear factor-κB (NF-κB) and p53, among others. We found that NSCLC cells transfected with wild-type PDK1 promoter-luciferase reporter construct showed decreased activity when exposed to ciglitazone (Figure 4B). As expected, metformin enhanced the inhibitory effect of ciglitazone (Figure 4B).Next, we assessed whether PPARγ activation played a role in mediating the effect of ciglitazone on PDK1 promoter activity. The effect of ciglitazone on inhibition of PDK1 promoter activity was not abrogated by PPARγ siRNA (Figure 4C, lower panel). Note that PPARγ siRNA blocked PPARγ protein expression (Figure 4C, upper panel). As expected, we found that compound C reduced the effect of ciglitazone on PDK1 promoter activity (Figure 4D).

### The role of transcription factor Egr-1 in mediating the effect of ciglitazone on expression of PDK1 and cell growth

**Figure 5:**
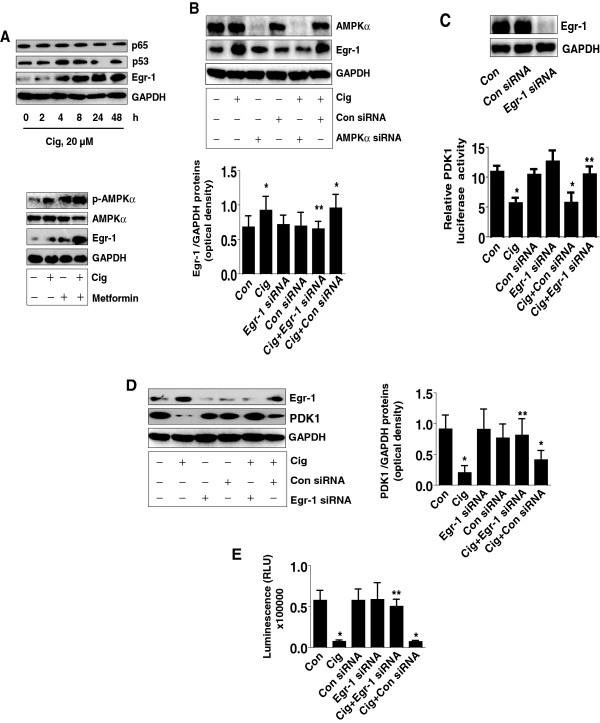
**Ciglitazone induces Egr-1 protein expression; silencing of Egr-1 abrogates the effect of ciglitazone on PDK1 promoter activity, protein expression and cell proliferation. A**, Cellular proteins were isolated from H1299 cells treated with ciglitazone (20 μM) for the indicated time period (upper panel) or with metformin (5 mM) for 24 h (lower panel). Afterwards, Western blot analyses were performed for detecting Egr-1, p65 and p53 proteins. **B**, H1299 cells were transfected with control or AMPKα siRNA (80 nM) for 30 h before exposing the cells to ciglitazone (20 μM) for an additional 24 h followed by Western blot. **C**, H1299 cells were transfected with control or Egr-1 siRNA (80 nM) together with a wild type PDK1 promoter construct for 30 h, then cells were exposed to ciglitazone (20 μM) for an additional 24 h. Insert shows the Western blot result for Egr-1 protein. **D**, H1299 cells were transfected with control or Egr-1 siRNA (80 nM) for 30 h before exposing the cells to ciglitazone (20 μM) for an additional 24 h, followed by Western blot. **E**, H1299 cells were transfected with control or Egr-1 siRNA (80 nM) for 30 h before exposure of the cells to ciglitazone (20 μM) for an additional 24 h. Afterwards, the luminescence of viable cells was detected using Cell Titer-Glo Luminescent Cell Viability Assay kit. The bars represent the mean ± SD of at least four independent experiments for each condition. *Indicates significant difference as compared to the control. **Indicates significance of combination treatment as compared with ciglitazone alone (P < 0.05).

We further tested the role of the transcription factors in mediating the effect of ciglitazone on PDK1 expression in human lung carcinoma cells. We showed that ciglitazone significantly induced the expression of Egr-1 protein in a time–dependent manner, while it had little effect on p65 and p53 (Figure 5A, upper panel). Note that a synergy was observed in the combination of ciglitazone and metformin treatment (Figure 5A lower panel). Interestingly, we also found that silencing of AMPKα abolished the effect of ciglitazone on Egr-1 protein expression, further suggesting the critical role of AMPKα activation in this process (Figure 5B). Next, we found that while cells transfected with Egr-1 siRNA slightly increased PDK1 promoter activity at baseline, it greatly antagonized the inhibitory effect of ciglitazone on PDK1 promoter activity. Note that the control siRNA had no effect (Figure 5C, lower panel). Egr-1 siRNA reduced the production of Egr-1 protein (Figure 5C, upper panel). Furthermore, it eliminated the ciglitazone-reduced PDK1 protein expression, whereas the control siRNA had no effect (Figure 5D). Consistent with these findings, we found that cells transfected with Egr-1 siRNA blocked the inhibitory effects of ciglitazone on cell growth (Figure 5E). The control siRNA had no effect.

**Figure 6:**
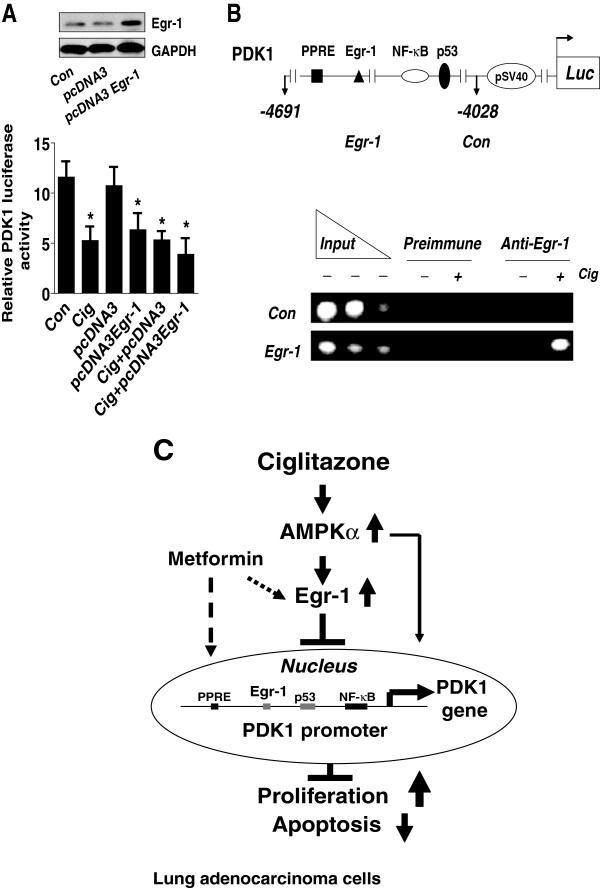
**Overexpression of Egr-1 reduces PDK1 promoter activity. A**, H1299 cells (1x10^5^ cells) were transfected with control and Egr-1 expression reporter constructs, and together with a wild type PDK1 promoter construct and an internal control Renilla Luciferase Reporter Vector as described in Material and Methods section for 24 h, then treated with ciglitazone (20 μM) for an additional 24 h. The insert in upper panel represents Western blot results for Egr-1 protein. **B**, H1299 cells were lysed after exposure of ciglitazone (20 μM) for 24 h, and nuclei were isolated and then sonicated. Chromatin from H1650 cells was immunoprecipitated using antibodies against Egr-1 protein or preimmune serum (pre-immune). PCR analysis using primers surrounding the Egr-1 site shows that this DNA sequence (−4392 to −4402 bp) is specifically immunoprecipitated indicating that Egr-1 binds to endogenous DNA sites in the PDK1 promoter. A non- Egr-1 sequence was used as control. Aliquots of the chromatin were also analyzed before immunoprecipitation (input). **C**, Diagram demonstrates that ciglitazone inhibits PDK1 expression through AMPKα-mediated induction of Egr-1 protein expression and Egr-1 protein binding to the DNA sequence in the *PDK1* gene promoter independent of PPARγ. Activation of AMPKα enhances the effect of ciglitazone on Egr-1 and PDK1 protein expression. In turn, this results in inhibition of NSCLC cell proliferation.

However, cells co-transfected with an Egr-1 expression vector showed little or no synergistic effect on PDK1 promoter activity (Figure 6A), suggesting the specificity of Egr-1. Next, by ChIP assays, we showed that ciglitazone induced Egr-1 protein binding to the Egr-1 DNA site in the *PDK1* gene promoter (Figure 6B).

## Discussion

The expression of PPARγ and the effects of PPARγ ligands on cell growth have been extensively studied in many carcinoma cell types including lung [6,20]. However, the exact mechanisms mediating the effects of PPARγ ligands on cell growth inhibition are not fully understood. We have found that ciglitazone, a TZD and one of the synthetic PPARγ ligands, inhibited growth and induced apoptosis of NSCLC cells through reduction of PDK1, a kinase and master regulator of a number of downstream signal cascades that are involved in suppression of apoptosis and promotion of tumor growth including lung cancer [13,21]. Inhibition of PDK1 in several cancer cells results in significant cell growth inhibition [14-17]. These observations suggest that PDK1 can be considered as a key mediator of neoplasia and a promising anticancer target. This result, together with the finding that exogenous PDK1 diminishes the effect of ciglitazone on cancer cell growth, suggests a critical role of PDK1 in this process.

The concentrations of ciglitazone used here, found significantly inhibition of PDK1 gene expression and cell growth , are consistent or even lower with those reported by others which showed a significant effect on cell growth and apoptosis at clinically achievable concentrations [22-25]. For example, ciglitazone inhibited the growth of androgen-dependent and -independent human prostate cancer cells starting at 10 and reached maximal at even 45 μM concentrations [23]. In another study, ciglitazone showed to significantly inhibit cell viability and proliferation of brain tumor stem cells starting at 5 and continued to 25 μM concentration [25].

We demonstrated that ciglitazone inhibited the expression of PDK1 protein independent of PPARγ signals. Consistent with this, the PPARγ-independent signals mediating the effects of PPARγ ligands on gene expression and cell proliferation including lung cancer have been shown in other studies [24,26,27] although PPARγ-dependent signals were observed [28,29]. We reasoned that targeting PDK1 may also involve such mechanisms by which ciglitazone inhibits NSCLC cell growth. Given the fact that silencing of the PPARγ gene by siRNA had no effect on blockage of the effect of ciglitazone on PDK1 promoter activity, additional experiments are required to explore the contributions of PPARγ-independent mechanisms in these processes.

Interestingly, PDK1 knockdown alone did not affect cell proliferation significantly. However, inhibition of PDK1 in the setting of ciglitazone treatment resulted in largely growth inhibition. This suggests that other factors are important for control of NSCLC cell proliferation. It also suggests that the growth inhibitory effects of ciglitazone may occur by concomitant actions on pathways other then PDK1. Report shown that ciglitazone exerts effects on several other targets that were implicated in control of lung cancer growth [30,31].

In this study, we showed that activation of AMPKα played a vital role in mediating the effect of ciglitazone on PDK1 expression. In addition, activation of AMPK enhanced the effect of ciglitazone on PDK1 expression and promoter activity. Data demonstrated that synthetic PPARγ ligands regulated several kinase signaling pathways including AMPK in different cells [32,33]. Activation or inactivation of AMPK has been shown to link synthetic PPARγ agonists-mediated signaling to the transcriptional regulation of genes that are crucial for cell growth inhibition [32,34]. Considering the recent data for the dual role of AMPK [9], we believed that more dedicated studies are required to further elucidate the biological function and relevant signaling of this kinase.

Having demonstrated the important role of PDK1, we further investigated whether the ciglitazone–mediated downregulation of PDK1 reflected inhibition of transactivation of the *PDK1* gene. Our results suggested that increased Egr-1 protein expression and binding to the upstream areas of *PDK1* gene promoter played an important role in mediating the effect of ciglitazone. Knockdown of Egr-1 abrogated the effect of ciglitazone on PDK1 expression and on cell proliferation, whereas overexpression of Egr-1 had no further effect of ciglitazone on PDK1 promoter activity confirming the inhibitory property of this transcription factor. It also suggested the specificity of Egr-1 played in this process. To our knowledge, the role of Egr-1 in regulation of PDK1 expression has never been reported. Egr-1 functions as a tumor suppressor in many cancers [10-12]. Loss of Egr-1 expression has been associated with invasion and anti-apoptotic events, whereas overexpression of Egr-1 suppressed the tumorigenicity and metastatic potential in several cancer cells including lung [34,35]. However, opposite role of Egr-1 were also found in several studies [36,37]. Thus Egr-1 is considered to play dual roles depending on the cell types and environment. One study showed that several PPARγ ligands including TZD induced the expression of Egr-1 through PPARγ-independent pathway in breast cancer cells [38]. Thus, other factors responsible for this effect (in addition to Egr-1) need further exploration. ChIP assays showed that Egr-1 protein occupancy of the Egr-1 sites in the upstream areas of *PDK1* gene promoter was enhanced by exposure of cells to ciglitazone. Further studies are required by site-directed mutagenesis experiments to confirm this. Moreover, the detail mechanisms responsible for the effect of metformin in this process needs to be determined.

## Conclusion

Our results demonstrate that ciglitazone inhibits PDK1 expression through AMPKα-mediated induction of Egr-1 protein expression and Egr-1 binding to specific DNA sequences in the *PDK1* gene promoter, which is independent of PPARγ activation. Activation of AMPKα by metformin enhances the effect of ciglitazone on Egr-1 and PDK1 protein expression. In turn, this leads to inhibition of NSCLC cell proliferation (Figure 6C). This study provides a novel mechanism by which the antidiabetic drug inhibits human lung cancer cell growth, and targeting the PDK1 may be a potential therapeutic strategy for inhibition of lung cancer growth.

## Materials and methods

### Culture and chemicals

The human NSCLC cell lines A549, H1650, PC9, H1975, H1299 and H358 were obtained from the Cell Line Bank at the Laboratory Animal Center of Sun Yat-sen University starting March 2012 (Guangzhou, China) and grown in RPMI-1640 medium supplemented with 10% heat-inactivated FBS, HEPES buffer, 50 IU/mL penicillin/streptomycin, and 1 μg amphotericin (complete medium). All cell lines have been tested and authenticated for absence of *Mycoplasma*, genotypes, drug response, and morphology using a commercially available kit (Invitrogen, Shanghai, China) in the Laboratory and Animal Center at Sun Yat-sen University in April 2010 and August 2012. Polyclonal antibodies specific for PDK1, phosphor-AMPKα (thr172) phosphor-p-SAPK/JNK (Thr183/Tyr185) and total AMPKα and SAPK/JNK were purchased from Cell Signaling (Beverly, MA). Polyclonal antibodies against PPARγ, AMPKα, p53, p65 and Egr-1 were purchased from Santa Cruz Biotechnology, Inc (Santa Cruz, CA, USA). Ciglitazone, SP600125, GW9662, compound C, metformin and other chemicals were purchased from Sigma Aldrich (St. Louis, MO, USA) unless otherwise indicated.

### Western blot analysis

Protein concentrations were determined by the Bio-Rad protein assay. Equal amounts of protein from whole cell lysates were solubilized in 2x SDS-sample buffer and separated on 10% SDS polyacrylamide gels. Membranes were incubated with antibodies against PDK1, PPARg phosphor-AMPKα (thr172) phosphor-p-SAPK/JNK (Thr183/Tyr185) and total AMPKα and SAPK/JNK, p53, p65 and Egr-1. The membranes were washed and incubated with incubation with a secondary goat antibody raised against rabbit IgG conjugated to horseradish peroxidase (Cell Signaling, Beverly, MA, USA). The membranes were washed again and transferred to freshly made ECL solution (Pierce, Rockford, IL, USA) for 1 min, and exposed to X-ray film.

### MTT cell viability assay

Cell viability was measured using the 3-(4, 5-dimethylthiazol-2- yl)-2, 5-diphenyltetrazolium bromide (MTT) assay. Briefly, NSCLC cells (5 × 10^3^ cells/well) were counted and seeded into a 96-well microtiterplate. The cells were treated with increasing concentrations of ciglitazone for up to 72 h. After incubation, 10 μL MTT solution (5 g/L) was added to each well and incubated at 37°C for an additional 4 h. Supernatant was removed, then 150 μL DMSO was added to each well and oscillated for 10 min. Absorbance at 490 nm was determined through the use of ELISA reader (Perkin Elmer, Victor X5, USA). Each experiment was repeated at least three times. Cell viability (%) was calculated as (absorbance of test sample/absorbance of control) × 100%.

### CellTiter-Glo luminescent cell viability assay

Human lung carcinoma cells were treated with compound C for 2 h or were transfected with control or Egr-1 siRNA or PDK1 expression vectors for 24 h before exposure of the cells to ciglitazone for an additional 24 h in 96-well plates in DMEM media with 0.5% FBS. Afterwards, cell viability was measured using the CellTiter-Glo Luminescent Cell Viability Assay kit (Promega, Shanghai, China) according to the instructions of the manufacturer.

### Detection of caspase-3/7 activity

Enzymatic activity of caspase-3/7 was measured using the Caspase-Glo 3/7 Assay kit (Promega, Shanghai, China) according to the manufacturer’s instruction. Briefly, NSCLC cells were seeded in 96-well plates and treated with or without 20 μM of ciglitazone for 48 h. Afterwards, the cells were lysed and incubated with 100 μL of Apo-ONE Caspase-3/7 reagent (substrate and buffer in the ratio of 1:100). After 1 h incubation in the dark at RT, the fluorescence of each well was measured at 485–520 nm by reading in an Epoch microplate reader (Biotek Instruments; Winooski, VT, USA).

### Treatment with AMPKα, PDK1, Egr-1 and PPARγ small interfering RNA (siRNA)

The siRNA human PDPK1 (EHU071261) was ordered from Sigma (Shanghai, China). The AMPKα (Cat No.sc-45312), Egr-1 siRNA (Cat No. sc-105070), PPARγ siRNA (Cat No. sc-29455), and control nonspecific siRNA oligonucleotides (Cat No. sc-37007) were purchased from Santa Cruz Biotechnology (Santa Cruz, CA, USA). For the transfection procedure, cells were grown to 60% confluence, and PDK1, Egr-1, and PPARγ and control siRNAs were transfected using the oligofectamine reagent (Invitrogen, Shanghai, China) according to the manufacturer’s instructions. Briefly, Lipofectamine was incubated with serum–free medium for 10 min., mixed with siRNA (80 nM), incubated for 20 min at room temperature before the mixture was diluted with medium and added to cells. After culturing for 30 h, cells were washed, resuspended in new culture media in the presence or absence of ciglitazone for an additional 24 h for Western Blot, cell growth, luciferase report assays and other experiments.

### Transient transfection assays

The original human PDK1 promoter construct was a gift from Dr. Michalik at the University of Lausanne and have been reported previously [39]. The PDK1 promoter construct contains approximately 1500 base pairs of the 5’ flanking region of the human PDK1 gene connected to the pGL3 basic luciferase reporter vector [39]. Briefly, NSCLC cells were seeded at a density of 5 × 10^5^ cells/well in 6-well dishes and grown to 50 –60% confluence. For each well, 2 μg of the control or PPRE X3-TK-luc reporter (Addgene, plasmid 1015) [40], above PDK1 plasmid DNA constructs, or overexpression of PDK1 (pDONR223-PDPK1) (Addgene plasmid 23801) [41] or Egr-1 expression vectors [obtained from Dr. Thomas Eling (NIEHS, USA) and have been reported previously] [42], with or without 0.2 μg of the internal control phRL-TK Renilla Luciferase Reporter Vector were co-transfected into the cells with the oligofectamine reagent (Invitrogen, Shanghai, China). In parallel experiments, NSCLC cells transfected with Egr-1, PPARγ, or control siRNAs (80 nM each) for 30 h followed by exposed the cells to ciglitazone for an additional 24 h. The preparation of cell extracts and measurement of luciferase activities were determined using the Dual-Luciferase Reporter Kit (Promega, Shanghai, China). Firefly luciferase activity was normalized with Renilla luciferase activity within each sample.

### Chromatin immunoprecipitation assay (ChIP)

ChIP assays were performed as described by other study [43]. Briefly, cells were incubated in 1% formaldehyde for 10 min at 37°C, quenched with 125 mmol/L glycine, lysed in SDS buffer with protease inhibitors (Roche), 0.5 mmol/L phenylmethyl-sulfonyl fluoride and sonicated. Fragmented chromatin was pre-cleared by adding salmon sperm-DNA/protein A-agarose beads. A portion of the supernatant was kept as “input” material. The remaining cleared chromatin was incubated overnight with or without 5 μg of anti-Egr-1 antibody or normal human IgG (Upstate Biotechnology, Shanghai, China). DNA (10 μg) from each immunoprecipitation was reserved for input controls. DNA was purified with QIAquick PCR purification column (QIAGEN Sciences, MA) and genomic sequences of interest were amplified by PCR using primers Egr-1 forward (−4392/-4402) 5′- GAGGGTGGACACAGTTGAGTCAG-3′ and reverse ′5-TGGACAACATTAGCAAGACCCTG-3′. A total of 2% of each IP was assayed by PCR using primers specific for the region of interest (218 bp).

### Statistical analysis

All experiments were repeated a minimum of three times. All data were expressed as means ± SD. and then processed using SPSS10.0 software. Statistical significance was determined with Student’s t test (two-tailed) comparison between two groups of data set. Asterisks shown in the figures indicate significant differences of experimental groups in comparison with the corresponding control condition (P < 0.05, see figure legends).

## Abbreviations

PPARγ: Peroxisome proliferator-activated receptors gamma; PDK1: Phosphoinositide-dependent protein kinase 1; Egr-1: Early growth response-1; siRNA: Small interfering RNA; ChIP: Chromatin immunoprecipitation assay; AMPKα: AMP-activated protein kinase alpha; SAPK/JNK: Stress-activated protein kinase/c-Jun N-terminal kinase; NSCLC: Non-small cell lung cancer; PPRE: PPAR response elements; NF-κB: Nuclear factor-κB; TZDs: Thiazolinediones.

## Competing interest

There is no potential conflict of interest or financial dependence regarding this publication.

## Authors’ contributions

SSH conceived of the study, participated in its design and coordination, and draft the manuscript. QT carried out the cell growth, siRNA, Western Blot assays, transfection and luciferase report assays. FZ participated in performed the cell viability, siRNA, transfection assays and protein expression experiments, SYZ involved in cell viability, protein expression and statistical analysis. JPC and ZYW provided agents, coordinated and critical read the manuscript. All authors read and approved the final manuscript.
